# Supplementary material for: Bayesian refinement of protein structures and ensembles against SAXS data using molecular dynamics
Source: PLoS Comput Biol. 2017 Oct 18;13(10):e1005800. doi: 10.1371/journal.pcbi.1005800 (PMC5662244; doi:10.1371/journal.pcbi.1005800)
Supplement: S1 Archive — (BZ2) [file pcbi.1005800.s011.tar.bz2 › gromacs_4.62_bayesian_saxs_refi/share/html/online/g_energy.html]

g\_energy


| g\_energy Main Table of Contents |

**VERSION 4.6  
Sat 19 Jan 2013**

---

### Description

g\_energy extracts energy components or distance restraint
data from an energy file. The user is prompted to interactively
select the desired energy terms.

Average, RMSD, and drift are calculated with full precision from the
simulation (see printed manual). Drift is calculated by performing
a least-squares fit of the data to a straight line. The reported total drift
is the difference of the fit at the first and last point.
An error estimate of the average is given based on a block averages
over 5 blocks using the full-precision averages. The error estimate
can be performed over multiple block lengths with the options
-nbmin and -nbmax.
**Note** that in most cases the energy files contains averages over all
MD steps, or over many more points than the number of frames in
energy file. This makes the g\_energy statistics output more accurate
than the .xvg output. When exact averages are not present in the energy
file, the statistics mentioned above are simply over the single, per-frame
energy values.

The term fluctuation gives the RMSD around the least-squares fit.

Some fluctuation-dependent properties can be calculated provided
the correct energy terms are selected, and that the command line option
-fluct\_props is given. The following properties
will be computed:  
Property Energy terms needed  
---------------------------------------------------  
Heat capacity C\_p (NPT sims): Enthalpy, Temp   
Heat capacity C\_v (NVT sims): Etot, Temp   
Thermal expansion coeff. (NPT): Enthalpy, Vol, Temp  
Isothermal compressibility: Vol, Temp   
Adiabatic bulk modulus: Vol, Temp   
---------------------------------------------------  
You always need to set the number of molecules -nmol.
The C\_p/C\_v computations do **not** include any corrections
for quantum effects. Use the g\_dos program if you need that (and you do).

When the -viol option is set, the time averaged
violations are plotted and the running time-averaged and
instantaneous sum of violations are recalculated. Additionally
running time-averaged and instantaneous distances between
selected pairs can be plotted with the -pairs option.

Options -ora, -ort, -oda, -odr and
-odt are used for analyzing orientation restraint data.
The first two options plot the orientation, the last three the
deviations of the orientations from the experimental values.
The options that end on an 'a' plot the average over time
as a function of restraint. The options that end on a 't'
prompt the user for restraint label numbers and plot the data
as a function of time. Option -odr plots the RMS
deviation as a function of restraint.
When the run used time or ensemble averaged orientation restraints,
option -orinst can be used to analyse the instantaneous,
not ensemble-averaged orientations and deviations instead of
the time and ensemble averages.

Option -oten plots the eigenvalues of the molecular order
tensor for each orientation restraint experiment. With option
-ovec also the eigenvectors are plotted.

Option -odh extracts and plots the free energy data
(Hamiltoian differences and/or the Hamiltonian derivative dhdl)
from the ener.edr file.

With -fee an estimate is calculated for the free-energy
difference with an ideal gas state:   
Δ A = A(N,V,T) - A\_idealgas(N,V,T) = kT ln()  
Δ G = G(N,p,T) - G\_idealgas(N,p,T) = kT ln()  
where k is Boltzmann's constant, T is set by -fetemp and
the average is over the ensemble (or time in a trajectory).
Note that this is in principle
only correct when averaging over the whole (Boltzmann) ensemble
and using the potential energy. This also allows for an entropy
estimate using:  
Δ S(N,V,T) = S(N,V,T) - S\_idealgas(N,V,T) = ( - Δ A)/T  
Δ S(N,p,T) = S(N,p,T) - S\_idealgas(N,p,T) = ( + pV - Δ G)/T

When a second energy file is specified (-f2), a free energy
difference is calculated   
 dF = -kT ln(\_A) ,
where E\_A and E\_B are the energies from the first and second energy
files, and the average is over the ensemble A. The running average
of the free energy difference is printed to a file specified by -ravg.
**Note** that the energies must both be calculated from the same trajectory.

### Files

| option | filename | type | description |
| --- | --- | --- | --- |
| **-f** | ener.edr | Input | Energy file |
| **-f2** | ener.edr | Input, Opt. | Energy file |
| **-s** | topol.tpr | Input, Opt. | Run input file: tpr tpb tpa |
| **-o** | energy.xvg | Output | xvgr/xmgr file |
| **-viol** | violaver.xvg | Output, Opt. | xvgr/xmgr file |
| **-pairs** | pairs.xvg | Output, Opt. | xvgr/xmgr file |
| **-ora** | orienta.xvg | Output, Opt. | xvgr/xmgr file |
| **-ort** | orientt.xvg | Output, Opt. | xvgr/xmgr file |
| **-oda** | orideva.xvg | Output, Opt. | xvgr/xmgr file |
| **-odr** | oridevr.xvg | Output, Opt. | xvgr/xmgr file |
| **-odt** | oridevt.xvg | Output, Opt. | xvgr/xmgr file |
| **-oten** | oriten.xvg | Output, Opt. | xvgr/xmgr file |
| **-corr** | enecorr.xvg | Output, Opt. | xvgr/xmgr file |
| **-vis** | visco.xvg | Output, Opt. | xvgr/xmgr file |
| **-ravg** | runavgdf.xvg | Output, Opt. | xvgr/xmgr file |
| **-odh** | dhdl.xvg | Output, Opt. | xvgr/xmgr file |

### Other options

| option | type | default | description |
| --- | --- | --- | --- |
| **-[no]h** | bool | no | Print help info and quit || **-[no]version** | bool | no | Print version info and quit || **-nice** | int | 19 | Set the nicelevel || **-b** | time | 0 | First frame (ps) to read from trajectory || **-e** | time | 0 | Last frame (ps) to read from trajectory || **-[no]w** | bool | no | View output .xvg, .xpm, .eps and .pdb files || **-xvg** | enum | xmgrace | xvg plot formatting: xmgrace, xmgr or none || **-[no]fee** | bool | no | Do a free energy estimate || **-fetemp** | real | 300 | Reference temperature for free energy calculation || **-zero** | real | 0 | Subtract a zero-point energy || **-[no]sum** | bool | no | Sum the energy terms selected rather than display them all || **-[no]dp** | bool | no | Print energies in high precision || **-nbmin** | int | 5 | Minimum number of blocks for error estimate || **-nbmax** | int | 5 | Maximum number of blocks for error estimate || **-[no]mutot** | bool | no | Compute the total dipole moment from the components || **-skip** | int | 0 | Skip number of frames between data points || **-[no]aver** | bool | no | Also print the exact average and rmsd stored in the energy frames (only when 1 term is requested) || **-nmol** | int | 1 | Number of molecules in your sample: the energies are divided by this number || **-[no]fluct\_props** | bool | no | Compute properties based on energy fluctuations, like heat capacity || **-[no]driftcorr** | bool | no | Useful only for calculations of fluctuation properties. The drift in the observables will be subtracted before computing the fluctuation properties. || **-[no]fluc** | bool | no | Calculate autocorrelation of energy fluctuations rather than energy itself || **-[no]orinst** | bool | no | Analyse instantaneous orientation data || **-[no]ovec** | bool | no | Also plot the eigenvectors with -oten || **-acflen** | int | -1 | Length of the ACF, default is half the number of frames || **-[no]normalize** | bool | yes | Normalize ACF || **-P** | enum | 0 | Order of Legendre polynomial for ACF (0 indicates none): 0, 1, 2 or 3 || **-fitfn** | enum | none | Fit function: none, exp, aexp, exp\_exp, vac, exp5, exp7, exp9 or erffit || **-ncskip** | int | 0 | Skip this many points in the output file of correlation functions || **-beginfit** | real | 0 | Time where to begin the exponential fit of the correlation function || **-endfit** | real | -1 | Time where to end the exponential fit of the correlation function, -1 is until the end |

---

http://www.gromacs.org  
gromacs@gromacs.org
